# Factors associated with dietary supplement use by people who exercise at gyms

**DOI:** 10.1590/S0034-8910.2015049005912

**Published:** 2015-09-23

**Authors:** Francisca Mirian Moura Lacerda, Wellington Roberto Gomes Carvalho, Elane Viana Hortegal, Nayra Anielly Lima Cabral, Helma Jane Ferreira Veloso

**Affiliations:** I Curso de Nutrição. Centro Universitário do Maranhão. São Luís, MA, Brasil; II Departamento de Educação Física. Universidade Federal do Maranhão. São Luís, MA, Brasil; III Departamento de Ciências Fisiológicas. Universidade Federal do Maranhão. São Luís, MA, Brasil

**Keywords:** Athletes, Dietary Supplements, utilization, Socioeconomic Factors, Cross-Sectional Studies

## Abstract

**OBJECTIVE:**

To assess the factors associated with the use of dietary supplements by people who exercise at gyms.

**METHODS:**

A cross-sectional study with a sample defined by convenience, considering the number of gyms registered in the *Conselho Regional de Educação Física* (Regional Council of Physical Education) of Sao Luis, MA, Northeastern Brazil, from July 2011 to July 2012. The final sample comprised 723 individuals who exercise at gyms. The dependent variable was supplement use, and the explanatory variables were length of time and motivation of the physical exercises, duration, goal and self-perception of training, weekly frequency of gym attendance, sex, age, educational attainment, self-perception of body weight, smoking and self-perception of diet. The association between variables was analysed by hierarchical Poisson regression based on a theoretical model.

**RESULTS:**

Supplement use was reported by 64.7% of the participants. Most of the sample was male (52.6%). The most frequent age group was 20 to 39 years (74.4%). Most participants (46.1%) had been exercising for over a year. The following variables were associated with supplement use: self-perceiving body weight as below ideal (p < 0.001), smoking (p < 0.001), exercising for 7 to 12 months (p = 0.028) or more than one year (p < 0.001), spending more than two hours at the gym (p = 0.051), and perceiving training as moderate (p = 0.024) or intense (p = 0.001).

**CONCLUSIONS:**

The use of supplements lacks proper professional guidance, being motivated by individuals unsatisfied with their low body weight and who perceive their workout as intense, which raises the need for monitoring this population.

## Abstract (pt)

**OBJETIVO:**

Analisar os fatores associados ao consumo de suplementos nutricionais por frequentadores de academias.

**MÉTODOS:**

Estudo transversal, com amostra por conveniência, considerando o número de academias cadastradas no Conselho Regional de Educação Física em São Luís, MA, no período de julho de 2011 a julho de 2012, composta por 723 praticantes de exercício físico. A variável dependente foi uso de suplementos e as variáveis explanatórias foram tempo e objetivo da prática de exercícios físicos, duração, objetivo e autopercepção do treino, frequência semanal na academia, sexo, idade, escolaridade, autopercepção do peso, fumo e autopercepção da alimentação. A associação das variáveis foram analisadas por regressão Poisson hierarquizada com base em modelo teórico.

**RESULTADOS:**

Relataram utilizar algum tipo de suplemento, 64,7% praticantes. A maioria da amostra foi composta por homens (52,6%). A faixa etária de 20 a 39 anos foi mais frequente (74,4%). A maioria dos entrevistados (46,1%) praticava exercício físico há mais de um ano. As seguintes variáveis apresentaram associação com consumo de suplementos: considerar seu próprio peso abaixo do ideal (p < 0,001), ser fumante (p < 0,001), praticar o exercício físico de 7 a 12 meses (p = 0,028) ou por mais de um ano (p = 0,001), permanência na academia por mais de duas horas (p = 0,051) e considerar o seu próprio treino moderado (p = 0,024) ou intenso (p = 0,001).

**CONCLUSÕES:**

O uso de suplementos não tem a devida orientação profissional, sendo motivada pelo individuo insatisfeito com o baixo peso corporal e que acredita ter um treino intenso, levantando à necessidade de acompanhamento desse público.

## INTRODUCTION

Sports nutrition seeks to develop dietary strategies to improve physical performance and attenuate the metabolic stress caused by exercise. With rare exceptions, supplements are unnecessary when a person’s diet is quantitatively and qualitatively adequate and accompanied by appropriate fluid intake.^1^

The energy and nutrient recommendations in the Dietary Reference Intakes (DRI) are adapted for healthy populations of the United States and Canada. However, because those international reference standards for nutrient intake apply to the requirements of both healthy and sedentary individuals, their indications for more physically active people are controversial.^20^

The energy intake recommended for sedentary individuals and individuals who exercise moderately is insufficient for athletes, as the latter are subjected to exhaustive training. However, it is unknown whether the DRI are adequate for people who exercise on a regular but noncompetitive basis.^21^

Supplement use is justified when dietary nutrient intake does not meet an individual’s requirements.^13^ Dietary supplements are usually offered in an untypical form of food, including tablets, capsules, powders, or pills. Although meal replacements should not occur without the advice of a physician or dietitian, this recommendation is not systematically followed by individuals who perform resistance training aimed at muscle hypertrophy.^a^

Studies conducted in Brazil^18^ ^,^ ^20^ point to an indiscriminate use of dietary supplements, particularly those including proteins and amino acids, by young nonathletes (15-25 years old) who perform resistance training. According to Santos and Santos,^20^ this use is facilitated by a lack of specific legislation banning the sale of such supplements without a prescription by a dietitian or physician nutrition specialist.

Therefore, the aim of this study was to analyse the factors associated with the use of dietary supplements by individuals who exercise at gyms.

## METHODS

This is a cross-sectional study conducted between 2011 and 2012 with individuals who exercise at gyms in Sao Luis, capital of Maranhao state, Northeastern Brazil. The area has a population of 1,014,837 inhabitants. The Index of Human Development (IHD) of Sao Luis is 0.768, the best in the state and the 249^th^ in Brazil.^b^

The study sample was established by convenience based on the number of gyms in Sao Luis, according to the *Conselho Regional de Educação Física* (CREF – Regional Council of Physical Education) of Maranhao. The CREF has 42 registered gyms.

The gyms were assessed regarding their current status of operation and available training modalities. The criteria to select gyms were: distribution over several neighbourhoods, number of different areas, and training modalities in which resistance training was mandatory. Gyms that offered training activities specific to a given age range or sex were excluded. The 21 gyms in Sao Luis that met the inclusion criteria were contacted. Of these, 17 agreed and four refused to participate.

First, gym owners were contacted with invitations to participate in the study. Next, data were collected on the number of members, fitness instructors available, sale of supplements on the premises, and available training modalities. Data were recorded on one standard form per gym. In a second stage, questionnaires were distributed directly to the gym users. Nutrition undergraduate students were trained to assist the principal investigator in data collection at the selected gyms. Gym users were randomly approached at the main entrances of the gyms at several times of the day and on several days of the week. The inclusion criteria for the gym users were: being a member of the gym and exercising two or more times per week. Inclusion criteria did not include sex, age or social class.

Interviewers approached people at the entrance of the gyms from Monday to Saturday, in rush hours (7 a.m. to 9 a.m. and 4 p.m. to 9 p.m.). Although 738 self-reported questionnaires were handed out, the answers to important items were lacking in some questionnaires, so incomplete questionnaires were excluded from the analysis. The final sample comprised 723 individuals who exercised at gyms.

The instrument used for data collection was a standardised self-reporting questionnaire comprising multiple-choice questions that were relevant to the study goals. It included items relative to the users’ lifestyles and intake of dietary supplements. The use of thermogenic, carbohydrate-rich or protein-rich supplements, micronutrients, isotonic beverages, meal replacement shakes, creatine, and herbal products was assessed. The participants could select more than one option; in these cases, the choices were recorded under more than one category of supplement. The source of the supplement indication could be: dietitians, physicians, fitness instructors, acquaintances or self-prescription.

A conceptual model with the following three blocks was used:

1. Demographic factors (age and sex);
2. Socioeconomic factors (occupation and education);
3. Habits and concepts (type of training, time practicing physical activity, weekly frequency, duration of workout, smoking, and self-perception of body weight).

Sex was considered a potential confounding factor, so the model was adjusted for this variable.

The second block included socioeconomic variables as intermediate factors in the theoretical model, since they can mediate association of variables related to habits and concepts with consumption of supplements.

In the third block, the habits and concepts were included, assuming that these variables may be influenced by socioeconomic and demographic factors.

Data were entered in duplicate, and both copies were compared to correct for potential errors. The statistical analysis included estimation of prevalence by the Chi-square test, used to analyse the differences between the observed and expected proportion, and Poisson regression to assess the associations of socioeconomic, demographic, and behavioural factors with supplement use. Crude analysis was done with a simple Poisson regression, and the non-adjusted Prevalence Ratio (PR) and 95% confidence interval (95%CI) were estimated. Hierarchical Poisson regression analyses were done based on a theoretical model. The significance level was set at p < 0.05. The statistical package Stata 10.0 (Stata Corp., College Station, United States) was used for statistical tests.

The dependent variable, supplement use, was categorized as “yes” when the participants were using it at the time of the interview and as “no” when they had only used it in the past. The explanatory variables included the length of time the user had been exercising (< 1 month; 1-6 months; 7-12 months; ≥ 1 year), educational attainment (elementary school; secondary school; bachelor’s degree; postgraduate degree), occupation (higher level jobs; technical jobs; economically inactive), weekly frequency at the gym, sex, age (adolescent: 12-19 years old; young adult: 20-39 years old; adult: 40 years old or older), self-perception of body weight according to Goston and Correia^7^ (above ideal; ideal; below ideal), smoking (smokers; non-smokers; former smokers), motivation for exercising (healthy lifestyle; weight loss; gaining muscle mass), self-perceived training intensity (mild; moderate; intense), and self-perception of diet (poor; good; optimal).

This study was conducted according to the guidelines laid down in the Declaration of Helsinki and approved by the Research Ethics Committee of Centro Universitário do Maranhão (Protocol 00316/2011, 5/30/2011). All participants signed an informed consent form.

## RESULTS

Supplement use was reported by 64.7% of the sample. Most participants were male (52.6%) and 20-39 years old (74.4%). Regarding occupation, 51.0% of the sample had higher level jobs, 22.0% had technical jobs, and 26.0% were economically inactive (Table 1). College graduates represented 41.1% of the participants, while 24.1% had pursued postgraduate studies, 30.0% were secondary school graduates, and 2.8% were elementary school graduates. Body weight was considered above ideal by 50.1% of the participants, ideal by 36.5%, and below ideal by 13.4% of the sample.

**Table 1:** Socioeconomic, demographic, and behavioural data of the study sample of exercising individuals. Sao Luis, MA, Northeasthern Brazil, 2011-2012.

Variable	n	%
Supplement use		
Yes	468	64.7
No	255	35.3
Sex		
Male	380	52.6
Female	343	47.4
Age (years)		
< 20	93	12.9
20 to 39	538	74.4
≥ 40	92	12.7
Occupation		
Economically inactive	188	26.0
Technical level	166	23.0
Higher level	369	51.0
Educational attainment		
Elementary school	20	2.8
Secondary school	217	30.0
Bachelor’s degree	302	43.1
Postgraduate education	134	24.1
Self-perception of body weight		
Above ideal	362	50.1
Ideal	264	36.5
Below ideal	97	13.4
Smoking		
Smoker	25	3.5
Nonsmoker	639	88.4
Former smoker	59	8.1
Time since started exercising		
Less than 1 month	188	16.3
1 to 6 months	201	27.8
7 months to 1 year	71	9.8
More than 1 year	333	46.1
Weekly training frequency		
< 3 times per week	76	10.5
3 to 5 times per week	528	73.0
> 5 times per week	119	16.5
Training length		
≤ 1 hour	212	29.3
> 1 and ≤ 2 hours	429	59.3
> 2 hours	82	11.4
Motivation to exercise		
Healthy lifestyle	648	89.6
Weight loss	23	3.2
Muscle mass gain	52	7.2
Training intensity		
Mild	66	9.1
Moderate	503	69.6
Intense	154	21.3
Self-perception of diet		
Poor	98	13.5
Good	550	76.1
Optimal	98	10.4

Only 3.5% of the participants were smokers, 88.4% were nonsmokers, and 8.1% were former smokers. A large fraction of participants (46.1%) had been exercising for more than one year, 9.8% had been exercising between seven months and one year, 27.8% between one and six months, and 16.3% had started exercising in the previous month. Most participants exercised three to five times per week (73.0%), for one to two hours per session. The most frequent motivation to exercise was a healthy lifestyle (89.6%), followed by a desire to gain muscle mass (7.2%) or to lose weight (3.2%). Of the participants, 9.1% classified their training intensity as mild, 69.6% as moderate, and 21.3% as intense. With regard to dietary self-perception, 10.4% reported having an optimal diet, 76.1% reported having a good diet, and 13.6% reported having a poor diet (Table 1).

Protein- and amino acid-based supplements were the most frequently used (12.0%), followed by micronutrient-rich (4.6%) and carbohydrate-rich (3.5%) products. Of the 64.7% of participants who reported using supplements, 34.4% made simultaneous use of multiple types (Table 2).

**Table 2:** Types of supplements used by the individuals who exercise in gyms. Sao Luis, MA, Northeastern Brazil, 2011-2012.

Supplement type	n	%
None	255	35.3
Protein	87	12.0
Shakes	11	1.5
Herbal agents	21	2.9
Creatine	11	1.5
Thermogenic	19	2.6
Carbohydrate-rich	25	3.5
Micronutrients	33	4.6
Isotonic	12	1.7
2 or more	249	34.4

Based on a crude analysis, the factors that had significant associations with supplement use were male sex (p = 0.013); secondary (p = 0.046) or graduate (p = 0.026) education; self-perception of weight as ideal (p = 0.006) or below ideal (p < 0.001); smoking (p < 0.001); exercising for one to six months (p = 0.005), seven months to one year (p = 0.006), or more than one year (p < 0.001); training three to five (p = 0.003) or more than five (p = 0.007) times per week; training more than two hours per session (p = 0.020); and self-perceived intensity as moderate (p = 0.001) or intense (p < 0.001) (Table 3).

**Table 3:** Crude analysis of the factors associated with supplement use by individuals who exercise in gyms. Sao Luis, MA, Northeastern Brazil, 2011-2012.

Variable	Supplement use (%)	PR	95%CI	p*
Sex				
Male	68.9	1.05	1.01;1.10	0.013
Female	60.1	1	Reference	
Age (years)				
< 20	61.3	1	Reference	
20 to 39	65.6	1.02	0.96;1.09	0.432
≥ 40	63.4	1.01	0.92;1.10	0.806
Occupation				
Technical level/Student	64.1	1	Reference	
Higher level	65.3	1.00	0.96;1.05	0.739
Educational attainment				
Elementary school	40.0	1	Reference	
Secondary school	64.5	1.17	1.00;1.37	0.046
Bachelor’s degree	67.3	1.19	1.02;1.39	0.026
Postgraduate education	63.2	1.16	0.99;1.36	0.060
Self-perception of body weight	41.0			
Above ideal	57.5	1	Reference	
Ideal	68.2	1.06	1.01;1.11	0.006
Below ideal	82.5	1.15	1.09;1.22	< 0.001
Smoking				
Nonsmoker	63.5	1	Reference	
Former smoker	67.8	1.02	0.95;1.10	0.500
Smoker	88.0	1.14	1.07;1.23	< 0.001
Time since started exercising				
< 1 month	45.0	1	Reference	
1 to 6 months	61.2	1.11	1.03;1.19	0.005
7 months to 1 year	64.8	1.13	1.03;1.24	0.006
> 1 year	73.9	1.19	1.12;1.28	< 0.001
Weekly training frequency				
< 3 times per week	47.4	1	Reference	
3 to 5 times per week	66.7	1.13	1.04;1.22	0.003
> 5 times per week	67.2	1.13	1.03;1.24	0.007
Training length				
≤ 1 hour	62.3	1	Reference	
> 1 and ≤ 2 hours	63.9	1.00	0.96;1.06	0.693
> 2 hours	75.6	1.08	1.01;1.15	0.020
Motivation to exercise				
Weight loss	60.9	1	Reference	
Healthy lifestyle	63.7	1.01	0.89;1.15	0.784
Muscle mass gain	78.9	1.11	0.96;1.27	0.135
Training modality				
Strength training	65.8	10.6	0.98;1.15	0.096
Others	55.4	1	Reference	
Training intensity				
Mild	40.9	1	Reference	
Moderate	63.2	1.15	1.06;1.26	0.001
Intense	79.9	1.27	1.16;1.39	< 0.001
Self-perception of diet				
Poor	57.1	1	Reference	
Good	65.8	1.05	0.98;1.12	0.115
Optimal	66.7	1.06	0.96;1.15	0.197

* Simple Poisson regression.

The use of dietary supplements was not significantly associated with age (p = 0.678), type of occupation (p = 0.941), motivation to exercise (p = 0.083), or dietary self-perception (p = 0.237) (Table 3).

The variables that remained in the final model were self-perception of weight as ideal (p < 0.001) and below the ideal (p < 0.001), smoking (p < 0.001), exercising for seven months to one year (p = 0.028) or more than one year (p < 0.001), training for more than two hours per session (p = 0.053), and self-perceived intensity as moderate (p = 0.024) or intense (p = 0.001) (Table 4).

**Table 4:** Hierarchical Poisson regression analysis of the factors associated with supplement use by individuals who exercise in gyms. Sao Luis, MA, Northeastern Brazil, 2011-2012.a

Group/Variable	Block 1^b^			Block 2^c^			Block 3^d^		
	PR	95%CI	p	PR	95%CI	p	PR	95%CI	p
Sex	1.05	1.01;1.10	0.009						
Educational attainment									
Elementary school					1				
Secondary school				1.17	1.00;1.37	0.046			
Bachelor’s degree				1.18	1.01;1.08	0.032			
Postgraduate education				1.16	0.98;1.36	0.074			
Self-perception of body weight									
Above ideal								1	
Ideal							1.05	0.99;1.12	0.064
Below ideal							1.12	1.06;1.19	0.000
Smoking									
Nonsmoker								1	
Former smoker							0.99	0.92;1.06	0.882
Smoker							1.14	1.06;1.22	0.000
Time since started exercising									
< 1 month								1	
1 to 6 months							1.07	0.99;1.15	0.060
7 months to 1 year							1.10	1.01;1.21	0.027
More than 1 year							1.13	1.05;1.21	0.001
Training length									
≤ 1 hour								1	
> 1 and ≤ 2 hours							0.99	0.93;1.03	0.684
> 2 hours							1.07	0.99;1.14	0.051
Training intensity									
Mild								1	
Moderate							1.10	1.01;1.21	0.024
Intense							1.17	1.07;1.29	0.001
Weekly frequency									
< 3 times per week								1	
3 to 5 times per week							1.07	0.99;1.16	0.063
> 5 times per week							1.06	0.97;1.16	0.188

PR: prevalence ratio

^a^ Only variables with p < 0.10 were shown in the table; the others were omitted. Results for each variable were shown only for the block in which the variable was entered first.

^b^ Block 1: adjusted for sex.

^c^ Block 2: adjusted for variable kept in block 1(sex) plus variables from block 2.

^d^ Block 3: adjusted for variable kept in block 1(sex), variable selected from block 2 (educational level) plus variables from block 3.

The most frequent sources of supplement indication were acquaintances (17.8%), self-prescription (15.8%), and physical education teachers (17.3%), whereas dietitians were only mentioned by 10.3% of the participants (Figure).

**Figure:**
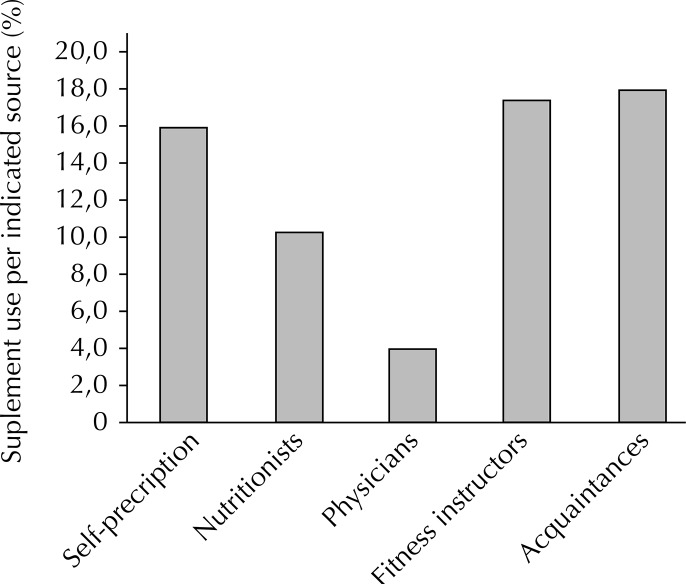
Source of supplement use indication. Sao Luis, MA, Northeastern Brazil, 2011-2012.

## DISCUSSION

This study showed the motivations that lead people to replace natural meals for dietary supplements without scientific evidence. The questions posed were appropriate to determine the causes of dietary supplement use by persons practicing physical exercise at gyms. In the current study, 64.7% of participants reported using dietary supplements. Such prevalence rate is greater than that described in the study of Goston and Correia,^7^ (36.8%) among exercisers in gyms in the city of Belo Horizonte, MG, Southeastern Brazil.^7^ Moreover, the authors found that use of supplements was associated with the people who needed them less, since their diet appeared concurrently to be good or excellent.^7^ Conner et al^4^ (2003) also found a similar result. In the present study, supplement use among exercisers in Sao Luis city was found to be way lower than rates observed in New York City (84.7%) and in Spain (56.1%).^15^ ^,^ ^16^ Exercisers in gyms constitute an important target for dietary supplement market.

Supplement use was frequent in the study sample but was not correlated with age, type of occupation, motivation for exercising, or dietary self-perception. Most participants were young adults, and the use of supplements did not exhibit significant differences as a function of age. The population of the study conducted in Belo Horizonte^7^ tended to be young, with an average age of 29 years, and supplement use was significantly more frequent among participants who were younger than 30 years of age.^7^ Male sex increased the odds of supplement use only on the crude analysis, and the association was lost after adjusting for other variables. The role of sex as a determinant of supplement use is still not clearly established. Study conducted in Botucatu, SP, Southeastern Brazil, also found greater supplement usage among males.^12^ However, the number of women in gyms seeking to achieve a well-defined body shape that is characterised by muscle hypertrophy (different from the traditional ideal) is increasing. This change might account for the similar frequency of supplement use among males and females in the present study.

A descriptive analysis showed that most participants (97.2%) had at least finished secondary school and 41.1% of them were college graduates. These results are in agreement with the findings of a study conducted with 309 gym users from Sao Paulo, in which 69.9% of participants had completed some higher education.^18^ Acknowledgement of the health benefits associated with exercise and the higher purchasing power of individuals with higher educational achievement might account for the greater frequency of these users.

Supplement use was independent of the participants’ educational levels in the adjusted analysis, which does not necessarily point to a neglect of health care because many athletes report using supplements based on the belief that supplements are a source of energy, prevent disease, help with weight loss, promote gains in muscle mass, or improve sports performance.^19^

A self-perception of body weight as ideal or below ideal increased the odds of supplement use. Participants who considered their body weight to be below ideal were the same participants who exhibited the highest frequency of supplement use. This finding cannot be explained on the grounds that such participants used supplements to improve a diet that was rated poor because the variable of self-perception of diet showed no association with supplement use. Other studies have shown that most supplement users are healthy and consider their dietary habits to be good or excellent.^7^ ^,^ ^17^ The association between self-perception of body weight and supplement use seems to be more strongly related to the desire to increase lean muscle mass than to a poor diet. Overvaluation of the anabolic properties of proteins and amino acids leads athletes to use dietary supplements.^11^ ^,^ ^14^ ^,^ ^18^

The association between smoking and supplement use is most likely explained by the fact that smokers are advised to ingest vitamin C because its antioxidant action neutralises the deleterious effects of the free radicals generated by smoking.^14^ Micronutrients were one of the most widely used supplement varieties in the present study.

Supplement use increased among participants with longer habits of exercising, possibly because of the influence of the gym environment and the amount of time spent with other gym users, which tend to stimulate the use of supplements. However, long regular exercise sessions also result in greater muscle hypertrophy, which cannot be attributed to supplement use by default. Another potential stimulus for supplement use is the performance plateau that occurs during an exercise regimen, which might compel individuals to resort to dietary supplements. The skeletal muscle is a malleable tissue that can have an altered phenotype in response to external stimuli, such as contractile activity and nutrient availability.^3^ The interaction between the exercise-induced response and nutrient availability has been known for several decades.^8^ ^,^ ^9^ This interaction encourages the industry to publicise the “magic” effects of supplements, promoting their use among athletes. The results from this survey thus underline the relevance of further investigations in the area of self-medication and the reasons for taking dietary supplements.

Training more than three times per week was associated with supplement use in the crude analysis. A study performed in Belo Horizonte, the capital of Minas Gerais state, found a strong correlation between training more than five times per week and supplement use, with a greater than threefold increase.^7^ Training more than two hours per session increased the odds of supplement use, similar to the findings of Goston and Correia.^7^ Another study, conducted in Sao Paulo, found an association between greater use of supplements, longer periods of regular exercise, and longer periods of time spent in the gym.^10^

Self-perception of training intensity as moderate or intense was strongly associated with supplement use, as did all variables related to training characteristics. More intense, more frequent, and longer training periods increased the odds of individuals using dietary supplements. Although they are widely available and sold on the gyms’ premises, inappropriate intake might harm users’ health.

The most frequent sources of supplement indication were acquaintances, fitness instructors, and self-prescription, whereas dietitians were only mentioned by 10.3% of the sample. In a study conducted in Belo Horizonte, most participants (55.0%) reported using dietary supplements without professional advice. Other studies found high percentages of supplement prescriptions by fitness instructors or personal trainers.^5^ ^,^ ^6^ These frequent prescriptions might arise because these professionals meet their customers at least twice a week, which is not the case for dietitians. Therefore, to achieve appropriate monitoring of individuals who exercise on a regular basis, sports dietitians must be members of the gym staff. Those professionals must dispel the widespread incorrect belief that nutrition science formally bans the use of dietary supplements; they can prescribe them when needed, based on the modality, frequency, and length of training within the broader scope of a balanced and individualised diet with full observance of ethical principles. To be successful without threatening the health of people who exercise on a regular basis, the prescription of dietary supplements must consider the individual’s diet.^2^ ^,^ ^17^

Self-perception of body weight was significantly correlated with supplement use because the participants who reported having ideal or below ideal weights were those who reported the highest use of dietary supplements. The length, frequency, and intensity of training were also significantly associated with dietary supplement use. These results, which are associated with a preference for protein-based supplements, suggest that the intended goal of supplement users is to increase muscle mass.

The convenience sample (with techniques of nonprobability sampling) and the self-reported weight (instead of measured directly) are limitations to the external validity of this study’s findings. However, our results could be extrapolated to other populations living in similar conditions.

In conclusion, self-perception of weight as ideal and below ideal, smoking, exercising for at least seven months, training for more than two hours per session, and self-perceived training intensity as moderate or intense were associated with consumption of supplements.

## Footnotes

a Conselho Federal de Nutricionistas. Resolução CFN nº 390, de 27 de outubro de 2006. Regulamenta a prescrição dietética de suplementos nutricionais pelo nutricionista e dá outras providências. *Diario Oficial Uniao*. 22 nov 2006; Seção I:104-5.

b Programa das Nações Unidas para o Desenvolvimento Humano; Instituto de Pesquisa Econômica Aplicada; Fundação João Pinheiro. Atlas do desenvolvimento humano no Brasil 2013. [cited 2015 Apr 28]. Available from: http://www.atlasbrasil.org.br/2013/perfil/sao-luis_ma

## References

[1] Alves LA, Biesek S, Alves LA, Guerra I (2005). Recursos ergogênicos nutricionais. Estratégias de nutrição e suplementação no esporte.

[2] Andrade LA, Braz VG, Nunes APO, Velutto JN, Mendes RR (2012). Consumo de suplementos alimentares por pacientes de uma clínica de nutrição esportiva de Sao Paulo. Rev Bras Cienc Mov.

[3] Coffey VG, Hawley JA (2007). The molecular bases of training adaptation. Sports Med.

[4] Conner M, Kirk SF, Cade KE, Barrett JH (2003). Environmental inﬂuences: factors inﬂuencing a woman’s decision to use dietary supplements. J Nutr.

[5] Dunn MS, Eddy JM, Wang MQ, Nagy S, Perko MA, Bartee RT (2001). The influence of significant others on attitudes, subjective norms and intentions regarding dietary supplement use among adolescent athletes. Adolescence.

[6] Frade RET, Stulbach T (2010). A importância da atuação do nutricionista em academias e clubes. Rev Nutr Pauta.

[7] Goston JL, Correia MI (2010). Intake of nutritional supplements among people exercising in gyms and influencing factors. Nutrition.

[8] Hawley JA, Gibala MJ, Bermon S (2007). Innovations in athletic preparation: role of substrate availability to modify training adaptation and performance. J Sports Sci.

[9] Hawley JA, Burke LM (2010). Carbohydrate availability and training adaptation: effects on cell metabolism. Exerc Sport Sci Rev.

[10] Hirschbruch MD, Fisberg M, Mochizuki L (2008). Consumo de suplementos por jovens freqüentadores de academias de ginástica em São Paulo. Rev Bras Med Esporte.

[11] Hoffman JR, Faigenbaum AD, Ratamess NA, Ross R, Kang J, Tenenbaum G (2008). Nutritional supplementation and anabolic steroid use in adolescents. Med Sci Sports Exerc.

[12] Junqueira JM, Maestá N, Sakzenian VM, Burini RC (2007). Uso de suplementos nutricionais e conhecimentos dietéticos de freqüentadores de academias de Botucatu/SP. Rev Nutr Pauta.

[13] (2009). Modificações dietéticas, reposição hídrica, suplementos alimentares e drogas: comprovação de ação ergogênica e potenciais riscos para a saúde. Rev Bras Med Esport.

[14] Morris K (2000). Vitamin C restores early coronary impairments in smokers. Lancet.

[15] Morrison LJ, Gizis F, Shorter B (2004). Prevalent use of dietary supplements among people who exercise at a commercial gym. Int J Sport Nutr Exerc Metab.

[16] Oliveira AJS, Miranda León MT, Guerra-Hernández E (2008). Estudio estadístico del consumo de suplementos nutricionales y dietéticos en gimnasios. A LAN.

[17] Oliveira ERM, Torres ZMC, Vieira RCS (2008). Importância dada aos nutricionistas na prática do exercício físico pelos praticantes de musculação em academias de Maceió, AL. Rev Bras Nutr Esporte.

[18] Pereira RF, Lajolo FM, Hirschbruch MD (2003). Consumo de suplementos por alunos de academias de ginástica em Sao Paulo. Rev Nutr.

[19] Rosenbloom CA, Jonnalagadda SS, Skinner R (2002). Nutrition knowledge of collegiate athletes in a Division I National Collegiate Athletic Association Institution. J Am Diet Assoc.

[20] Santos MAA, Santos RP (2002). Uso de suplementos alimentares como forma de melhorar a performance nos programas de exercício físico em academias de ginástica. Rev Paul Educ Fis.

[21] Steyn NP, Labadarios D, Nel JH, Heidi-Lee R (2005). Development and validation of a questionnaire to test knowledge and practices of dietitians regarding dietary supplements. Nutrition.

